# Blue Light‐Induced, Dosed Protein Expression of Active BDNF in Human Cells Using the Optogenetic CRY2/CIB System

**DOI:** 10.1002/biot.202400384

**Published:** 2024-12-26

**Authors:** Sina Christoffers, Nina Wichert, Elena Wiebe, Maria Leilani Torres‐Mapa, Madeleine Goblet, Jennifer Harre, Odett Kaiser, Marc‐Nils Wahalla, Holger Blume, Alexander Heisterkamp, Athanasia Warnecke, Cornelia Blume

**Affiliations:** ^1^ Institute of Technical Chemistry Leibniz University Hannover Hannover Germany; ^2^ Cluster of Excellence Hearing4all Hannover Germany; ^3^ Institute of Quantum Optics Leibniz University Hannover Hannover Germany; ^4^ Department of Otorhinolaryngology Hannover Medical School Hannover Germany; ^5^ Institute of Microelectronic Systems Leibniz University Hannover Hannover Germany

**Keywords:** BDNF, cochlea implant, gene expression, HEK293, neurotrophins, spiral ganglion neurons

## Abstract

The use of optogenetic tools offers an excellent method for spatially and temporally regulated gene and protein expression in cell therapeutic approaches. This could be useful as a concomitant therapeutic measure, especially in small body compartments such as the inner ear, for example, during cochlea implantation, to enhance neuronal cell survival and function. Here, we used the blue light activatable CRY2/CIB system to induce transcription of brain‐derived neurotrophic factor (BDNF) in human cells. Transfection with three plasmids, encoding for the optogenetic system and the target, as well as illumination protocols were optimized with luciferase as a reporter to achieve the highest protein expression in human embryonic kidney cells 293. Illumination was performed either with a light‐emitting diode or with a scanning laser setup. The optimized protocols were applied for the production of BDNF. We could demonstrate a 64.7‐fold increase of BNDF expression upon light induction compared to the basal level. Light‐induced BDNF was biologically active and enhanced survival and neurite growth of spiral ganglion neurons. The optogenetic approach can be transferred to autologous cell systems, such as bone marrow‐derived mesenchymal stem cells, and thus represents the first optogenetic neurotrophic therapy for the inner ear.

## Graphical Abstract and Lay Summary

The study investigates the use of optogenetic tools to control gene expression in human cells, with a particular focus on therapeutic measures in the inner ear, such as cochlear implant surgery. Using the CRY2/CIB light‐sensitive system, the researchers were able to effectively regulate BDNF expression in a standardized manner using both LEDs and lasers, resulting in improved survival and growth of cochlear spiral ganglion neurons.

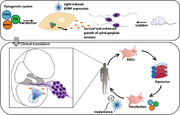

**Abbreviations**
BDNF: brain‐derived neurotrophic factor
CBCR: cyanobacteriochrome
CI: cochlear implant
CIB: cryptochrome‐interacting basic‐helix‐loop‐helix
CRY2: cryptochrome 2
CTB: Cell Titer‐Blue
DFG: DreamFectTM Gold
Goi: gene of interest
HEK: human embryonic kidney cells
LED: light‐emitting diode
LOV: light‐oxygen‐voltage
MSC: mesenchymal stem cells
PhyB: phytochrome B
PIF: phytochrome‐interacting factor
SGN: spiral ganglion neurons

## 1 Introduction

The field of optogenetics is rapidly growing in relevance and number of developed tools. Originating in the manipulation of light‐responsive ion channels, optogenetic systems have progressed to regulate gene expression. The use of light to induce cellular functions offers several advantages over chemically inducible systems such as noninvasiveness and a high temporal and spatial resolution [1]. Our work is focused on the optogenetic control of gene expression in human cells as suitable models for clinical applications in delimited target regions such as the inner ear as a concomitant therapeutic measure, for example, during cochlear implant (CI) surgery. Here, optogenetic gene regulation offers an excellent method for spatiotemporally regulated protein expression, for example, by the production of neurotrophic or anti‐inflammatory factors to enhance neuronal survival [2]. Suitable optogenetic systems consist of a split transcription factor, which is combined into a functional unit under light activation of a chromophore to activate the promoter of a specific target sequence. This induces protein biosynthesis of, for example, neuroprotective factors such as brain‐derived neurotrophic factor (BDNF). In the work presented here, a blue light (450 nm) inducible optogenetic system with cryptochrome circadian regulator 2 (CRY2) was used to trigger gene and protein expression of BDNF in human HEK293 cells.

**FIGURE 1:**
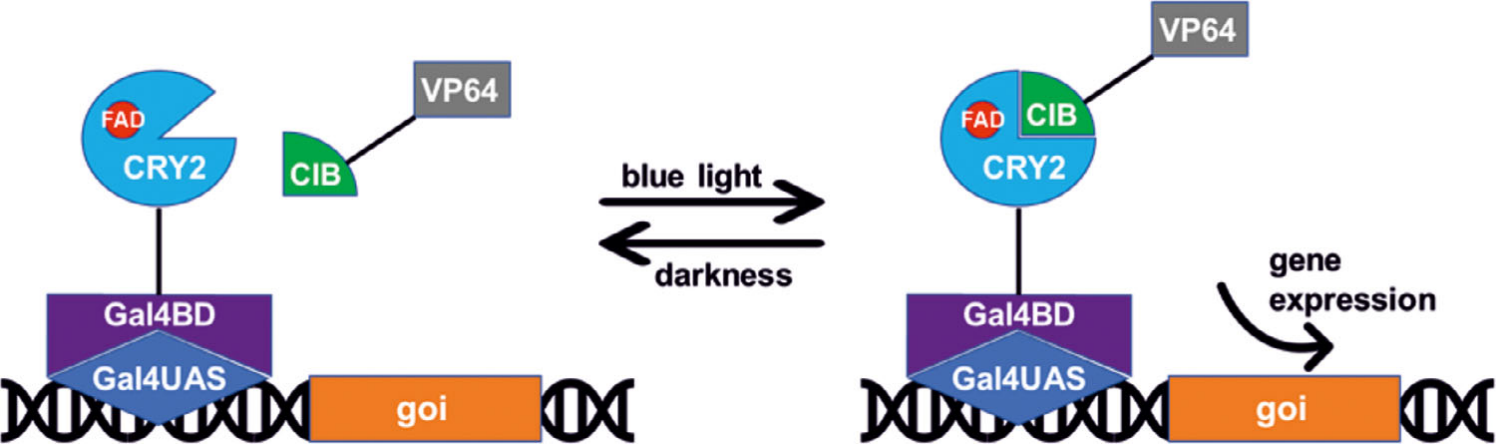
Working principle of the optogenetic CRY2/CIB system. CRY2 binds upstream of the gene of interest (goi) by a fused Gal4 DNA‐binding domain (Gal4BD). The blue light absorption of the non‐covalently linked chromophore flavin‐adenine‐dinucleotide (FAD) induces a conformational change of CRY2, enabling the binding of cryptochrome‐interacting basic‐helix‐loop‐helix (CIB) protein fused to the transcriptional activator VP64 (four tandem copies of VP16). VP64 activates transcription of the goi. Passive deactivation occurs in the dark.

The used CRY2/CIB system induces gene expression through a combination of three plasmids hosting the genetic information for the gene sections CRY2‐GalΔDD, CIBN‐VP64, and Gal4‐UAS‐BDNF/Luc (Figure 1). The photoreceptor CRY2 absorbs blue light after binding the endogenously expressed chromophore flavin‐adenine‐dinucleotide (FAD) that undergoes a conformational change, allowing it to dimerize to the N‐terminal domain of cryptochrome‐interacting basic‐helix‐loop‐helix (CIB). The Gal4BD (BD: binding domain) bound to CRY2 is the binding domain for the Gal4UAS (UAS: upstream activating sequence). After the Gal4BD has bound to the Gal4UAS, the CIB‐fused transcription factor VP64 ultimately activates the expression of the respective gene of interest with consequent protein expression [3, 4, 5]. In the dark, CIB detaches from CRY2 within a few minutes [6].

A lot of research is done in developing optimized optogenetic tools for gene regulation in clinical applications, which differ in activation wavelength, chromophore, reversibility, and deactivation time [2]. Since every system has its advantages and disadvantages, it has to be chosen carefully depending on the desired application. Common optogenetic systems used in mammalian cells, besides the CRY2/CIB system, are the blue light‐oxygen‐voltage (LOV) system [4, 5, 7] and the red light phytochrome B/phytochrome‐interacting factor (PhyB/PIF) system [8, 9, 10, 11]. Newer systems, on the other hand, are based on PhyA (REDMAP [12]) or on cyanobacteriochrome (CBCR). The PhyA and PhyB systems are optogenetic switches that are activated with red light and deactivated with far‐red light, which is advantageous over blue light in terms of tissue penetration depth and reduced phototoxicity effects due to the longer wavelength and less energy. The CBCR‐based light‐inducible dimers (BICYCLs [13]) are very small optogenetic switches that are either activated with green or with red light. However, all these above mentioned red light systems rely on the chromophore phycocyanobilin that is not endogenously expressed in mammalian cells and would thereby need to be injected [14] or the enzymes needed for its biosynthesis would have to be co‐expressed [9, 15], which would significantly complicate its use for a therapeutic application. In the first case, injections for sustained application of this cofactor would have to be performed through the round window of the inner ear, however, this invasive procedure would always be a risk to damaging the inner ear structures and possibly provoke leakage of fluids. In the second case, the additional transfection with, for example an AAV vector, would negate the advantage of a small optogenetic system. Therefore, we decided to use the blue light optogenetic system CRY2/CIB, which requires no exogenous chromophore.

BDNF is an excellent target protein for research and various therapeutic approaches, since it plays an important role in the survival and growth of neurons by binding to tyrosine kinase B, thereby activating diverse signal transduction cascades (IRS1/2, PI3K, and Akt). BDNF also serves as a regulatory factor for neurotransmitters [16, 17, 18]. A temporally and spatially controllable BDNF release could help neurons recover after surgery [19, 20] and thus represents a new endogenously light‐inducible pharmacological principle. To promote the growth of spiral ganglion neurons (SGNs) toward a cochlea implant (CI), optogenetically activatable cells could be placed next to a CI together with µLEDs or an optical fiber for laser delivery during surgery. This approach would increase the electrical resonance by stabilizing SGNs in the inner ear and enhance electrical coupling.

Both the chemical transfection of HEK293 cells with this CRY2 system and the illumination modes were optimized for high gene and protein expression. Since luciferase assays are highly sensitive, easy to perform, and reproducible, optimization experiments were performed with the luciferase reporter, and parameters relevant to the efficiency of chemical transfection, such as transfection reagent, plasmid ratio, and plasmid quantity were adjusted. In addition, the illumination time was varied. The optimized parameters using the LED illumination protocol were also implemented using a focused laser beam to enable a more spatially resolved protein synthesis in the targeted cells. After the successful delivery of the *BDNF* sequence into the *CRY2*/*CIB* plasmid system, the established transfection and illumination protocols enabled light‐pulse‐dependent BDNF protein expression. Its biological activity was demonstrated in SGN survival and neurite growth.

## 2 Results

### 2.1 LED Illumination and Phototoxicity

For the illumination experiments, a custom build LED chamber was developed to control the illumination time and intensity, as well as the temperature, CO_2_ content, and humidity for optimal cultivation conditions (Figure S1).

Since blue light exposure can potentially induce cell stress and death, light pulses of 20 s every 80 s (0.8 mW cm^−2^, 465 nm) were applied instead of constant illumination. This light intensity is in a completely different order of magnitude than, for example, the artificial light in our laboratory with an intensity of only 0.009 mW cm^2^. Thus, to assess the viability of HEK293 cells, the cells were illuminated 48 h after cell seeding for a period of 12 h, and a Cell Titer‐Blue (CTB) assay was performed after an additional resting period of 12 h. Illuminated cells showed only a slight decrease in viability with 87.3% ± 3.8% (mean ± sd, *N* = 3, *n* = 4) compared to the control of non‐illuminated cells. According to DIN EN ISO 10993‐5, a minimum cell viability of 70% is considered as nontoxic.

BDNF is a secretory protein, carrying the translocation signal in the first 18 amino acids of the gene sequence. To further confirm the pathway of the BDNF secretion based on the Golgi apparatus and not on membrane leakage due to any phototoxic effect, transfected cells were treated with 5 µg mL^−1^ Brefeldin A, an exocytosis inhibitor, for 6 h during illumination. Brefeldin A treatment reduced BDNF concentration in the medium from 47.17 to 1.05 ng mL^−1^ (Figure S2).

### 2.2 Optimized Transfection with Luciferase as Target Protein using LED Illumination

Gene expression and especially co‐transfection efficiency of different plasmids at the same time strongly depends on the cell type and transfection method. Therefore, we first optimized the gene transfer of the optogenetic system into HEK293 cells via lipofection with DreamFect Gold (DFG) for optimal protein synthesis using luciferase as a reporter protein due to its low detection limit and fast degradation kinetic to avoid saturation of the activity.

**FIGURE 2:**
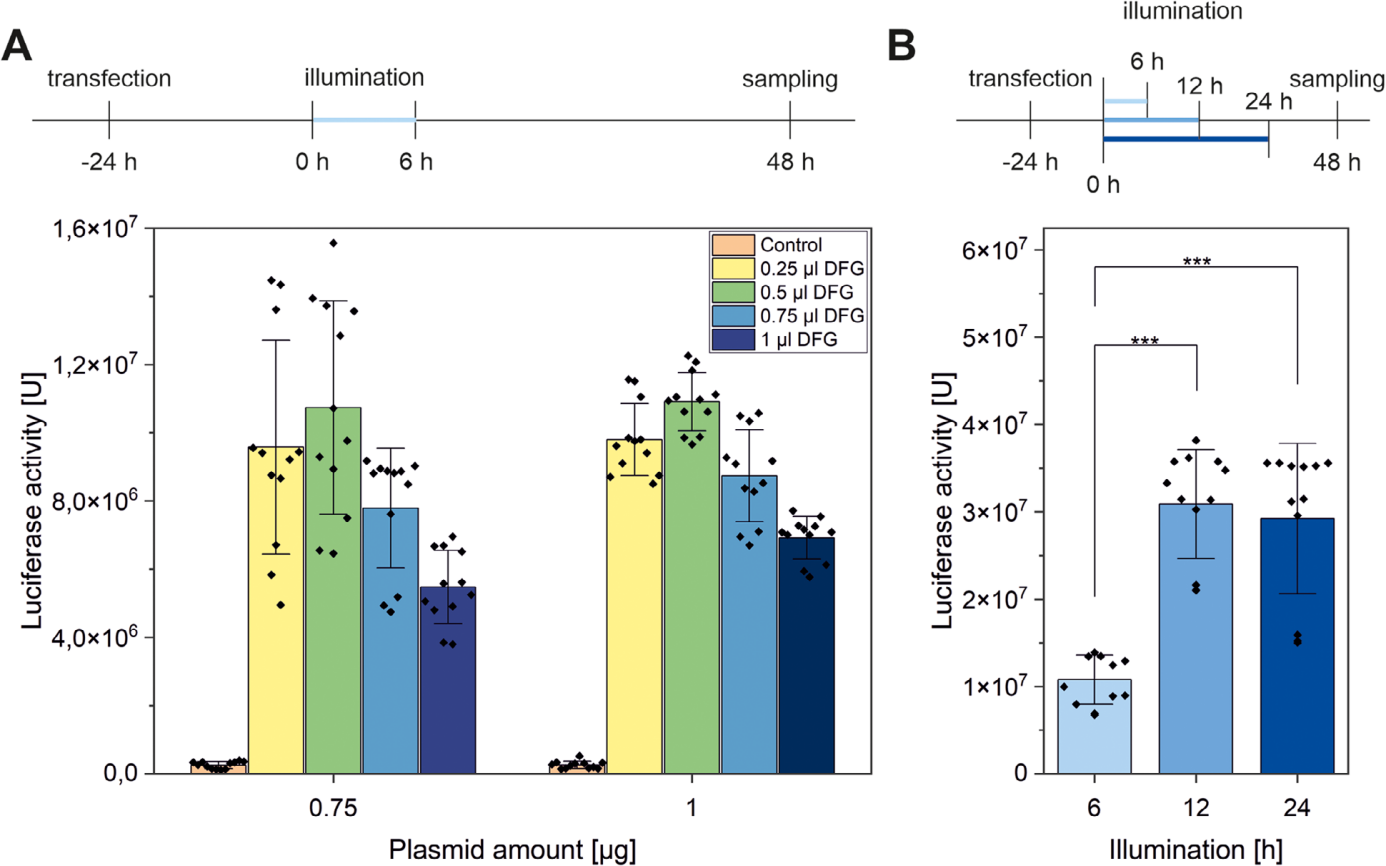
Assessment of optimal transfection conditions on optogenetic protein translation using the luciferase‐reporter system. (A) Effect of the total plasmid amount and the quantity of the DreamFect Gold (DFG) lipofection reagent on the luciferase activity after pulsed LED‐illumination for 6 h (20 s on/60 s off, 0.8 mW cm^−2^, 465 nm), measured 72 h after transfection of HEK293 cells with a fluorimetric luciferase assay. The total plasmid amount contained CRY‐GalΔDD, CIB‐VP64, and pGL2‐GAL4‐UAS‐Luc in a fixed ratio of 1:1:4. Non‐illuminated cells were used as a control. (B) Effect of the total duration of pulsed LED‐illumination (20 s on/60 s off, 0.8 mW cm^−2^) on the luciferase activity. HEK293 cells were transfected with 0.25 µL DFG and a total plasmid amount of 0.75 µg per four wells (mean ± sd, *N* = 3, *n* = 4, *p ≤ 0.05, **p ≤ 0.01, one‐way ANOVA with Tukey).

Twenty‐four hours after transfection, the cells were illuminated for 6 h, and luciferase activity was determined 48 h later. Non‐illuminated cells were used as a control, and the spontaneous luciferase activity was negligibly small. Concerning the transfection, we adjusted the amount of total DNA and the volume of the lipofection reagent. A volume of 0.5 µL DFG per well gave the best result for both plasmid amounts of 0.75 and 1 µg (Figure 2A). Another important parameter for the optogenetic control of gene regulation is the light dosage. An increase from 6 to 12 h of illumination led to a significant increase of luciferase activity by 2.5‐fold (Figure 2B). However, a further increase to 24 h did not lead to a higher output. At this point, the equilibrium reaction of the photoreceptor may be totally shifted to the activated state. Therefore, the enzyme activity and thus substrate conversion by luciferase in the CRY2/CIB system can very easily be controlled by adjusting the length of illumination or the light intensity. A lower light intensity of 0.45 mW cm^−2^ leads also to a 50% reduction of luciferase activity (Figure S3).

In all further experiments, the optimized conditions with 0.5 µL transfection reagent per four wells, the lower plasmid amount of 0.75 µg to conserve the stock, and an illumination time of 12 h was used.

### 2.3 Effect of Plasmid Ratio on BDNF Expression Using LED Illumination

**FIGURE 3:**
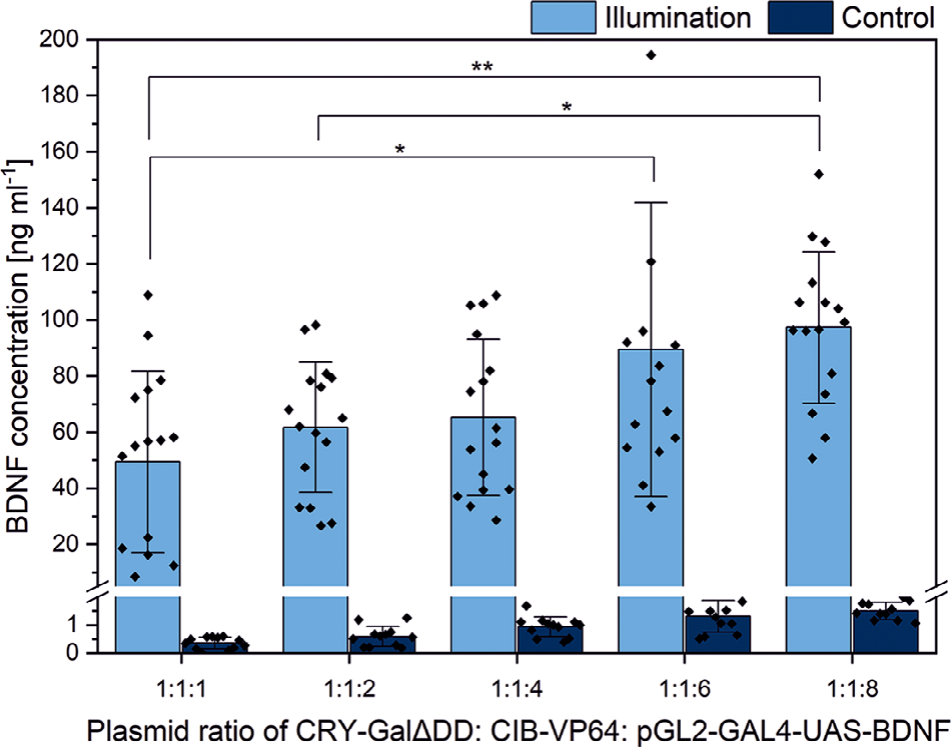
Effect of different tested plasmid ratios of CRY‐GalΔDD, CIB‐VP64, and pGL2‐GAL4‐UAS‐BDNF on BDNF protein expression using a total quantity of 0.75 µg plasmid DNA and a pulsed LED‐illumination for 12 h (20 s on/80 s off, 0.8 mW cm^−2^, 465 nm). Control cells were kept in the dark (mean ± sd, *N* = 3, *n* = 4, *p ≤ 0.05, **p ≤ 0.01, one‐way ANOVA with Tukey).

To achieve light‐induced BDNF protein expression, we replaced luciferase with *BDNF* downstream of Gal4UAS to obtain the plasmid pGL2‐GAL4‐UAS‐BDNF. We further optimized the transfection protocol by determining the effect of the plasmid ratio of CRY‐GalΔDD, CIB‐VP64, and pGL2‐GAL4‐UAS‐BDNF on the BDNF protein expression. HEK293 cells were illuminated with pulsed light, and cells that were kept in the dark served as a control. An increased quantity of the target plasmid (plasmid containing the *BDNF* sequence) led to an increase in the overall BDNF protein production but also in the leakage of the system (Figure 3). Illuminated cells had a 64.7‐fold increase over the control when the target plasmid was eight times higher than the other two plasmids (1:1:8). Moreover, BDNF expression was reversible and could be induced consecutively with the same cells over a total of 3 d in response to an illumination of 12 h once every day (Figure S4).

We hypothesize that the increased availability of the BDNF plasmid compared to CRY2 and CIB has a positive effect on the probability of random collision and thus on the binding of Gal4 to the binding motif upstream of BDNF, resulting in enhanced BDNF transcription and subsequent translation. Since we were interested in the highest protein production, a plasmid ratio of 1:1:8 of CRY‐GalΔDD, CIB‐VP64, and pGL2‐GAL4‐UAS‐BDNF was used for all further approaches.

### 2.4 Transcriptions and Protein Expression Analyses Using LED Illumination

**FIGURE 4:**
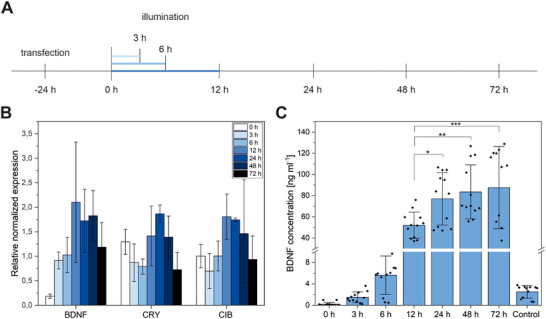
Time kinetics of normalized mRNA and protein expression. (A) Samples were analyzed before (0 h), during (3 and 6 h), and after (12, 24, 48, and 72 h) illumination. (B) Gene expression of *BDNF* and the optogenetic components *CRY2* and *CIB* relative to the housekeeping genes *RPL4, PPIA*, and *B2* *M* and normalized to the control, taken after 72 h. Cells were lysed and isolated RNA was translated to cDNA for qPCR analysis (mean ± sd, *N* = 2–3, *n* = 4). (C) Protein expression of BDNF in HEK293 cells. Supernatants were taken and BDNF protein expression was determined by ELISA (mean ± sd, *N* = 3, *n* = 4, *p ≤ 0.05, **p ≤ 0.01, ***p ≤ 0.001, one‐way ANOVA with Tukey, for higher clarity significances not shown for 0, 3, and 6 h).

*CRY2*/*CIB* and *BDNF* gene transcription and BDNF protein translation on the same cells were analyzed at different time points by qPCR or ELISA assay, respectively. Gene transcription was normalized to control cells that were kept in the dark (Figure 4A). *CRY2* and *CIB* were expressed at nearly the same level, reaching a maximum at 12–24 h after the start of illumination, and subsequently declined within the following 48 h (Figure 4B). In turn, the *BDNF* expression is only above the background signal after 6 h of illumination and increases 2‐fold after 12 h of illumination with a slower decline 72 h after the start of illumination. Protein translation and subsequent secretion increase strongly after 12 h of illumination (Figure 4C). The highest measured BDNF concentration was at 72 h after illumination but without a significant increase over 24 h or 48 h.

### 2.5 Light‐Induced BDNF Synthesis via Laser

**FIGURE 5:**
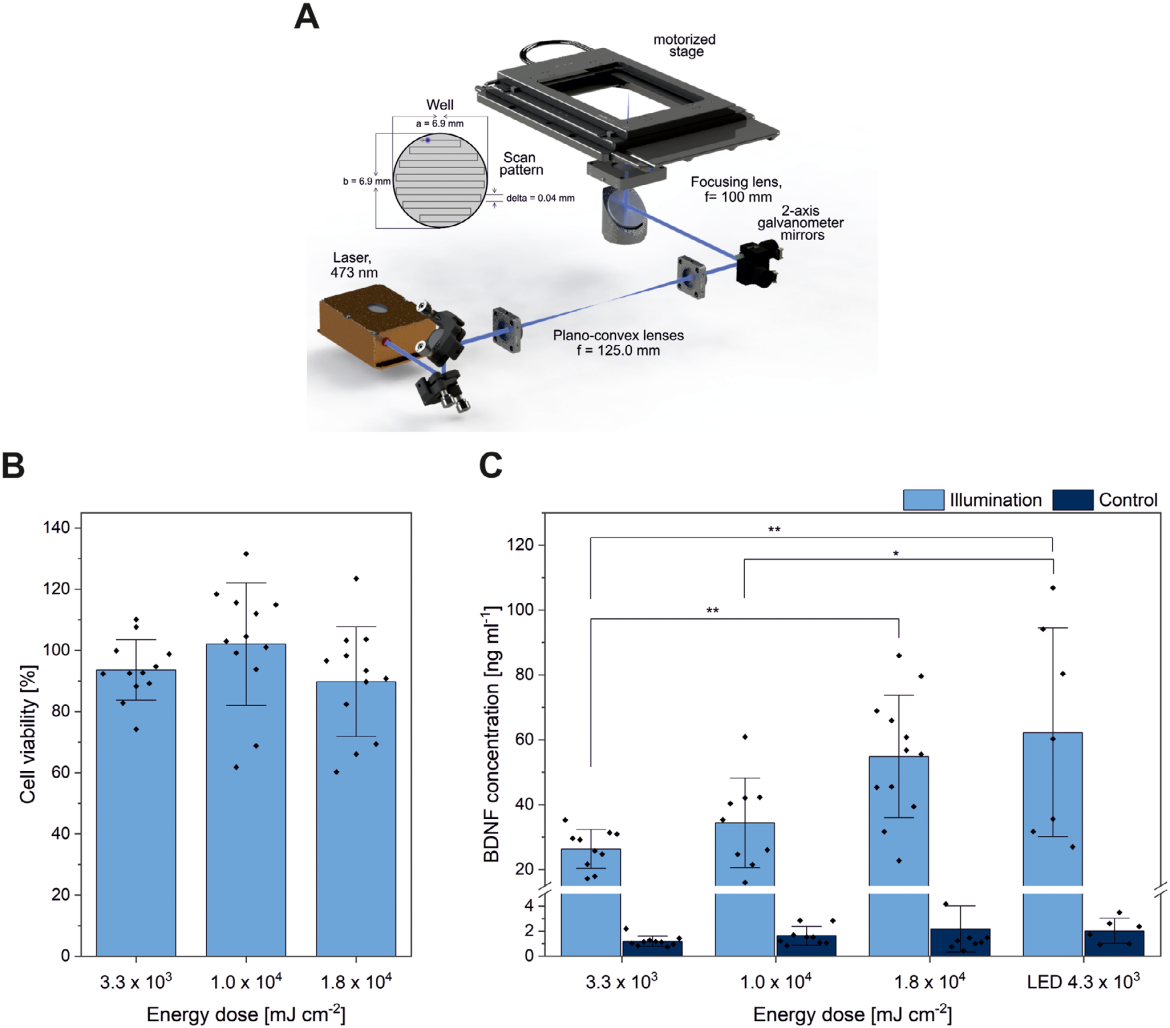
Laser set‐up and light‐induced BDNF production. (A) Laser setup and scanning pattern. The laser is reflected and guided by mirrors and optics onto a set of two galvo‐scanning mirrors. Another mirror reflects the light upwards and through a focusing lens from below onto the well plate. The laser scans the well with 35 mm s^−1^ in the shown pattern. (B) Influence of blue laser light over 6 h, using a meandering laser beam with a scanning speed of 35 mm s^−1^ and different energy doses (3.3 × 10^3^, 1.0 × 10^4^, and 1.8 × 10^4^ mJ cm^−2^) on the viability of HEK293 cells, measured with CTB assay. Illuminated cells were normalized to the control of non‐illuminated cells (mean ± sd, *N* = 3, *n* = 4). (C) Protein expression of BDNF after laser illumination for 6 h using aforementioned conditions (mean ± sd, *N* = 3, *n* = 4, *p ≤ 0.05, **p ≤ 0.01, one‐way ANOVA with Tukey).

Although LEDs have been used for most optogenetic experiments on cell monolayers and tissues, a focused laser spot offers a higher degree of spatial control for localized optogenetic activation of cells compared to LEDs and is also conceivable for a clinical approach. Therefore, we designed a laser scanning setup to induce BDNF expression (Figure 5A). For every experiment, four wells were illuminated with a power of 0.6, 1.9, and 3.3 mW for a duration of 6 h. For the laser scanning speed of 35 mm s^−1^, the total scanning time for the four wells including the return of the motorized stage to its initial position was measured to be 113 s, which sums up to about 191 line scans per well in 6 h.

To provide comparable illumination parameters for both systems, we quantified the accumulated energy dose (mJ cm^−2^) considering that LED illuminates all cells in the well simultaneously, whereas the laser scanning system has a focal spot that illuminates each region in the well sequentially for a short time. We first assessed the possible phototoxicity of the different laser energy doses on the cell viability of HEK293 cells with a CTB assay (Figure 5B). Energy doses up to 1.8 × 10^4^ mJ cm^−2^ showed no significant decrease in cell viability compared to the control. BDNF protein concentration increases as a function of energy dose. For the highest laser energy dose, a more than 25‐fold increase in BDNF over the control was observed. BDNF concentration reaches nearly the same level compared to LED for 6 h illumination with a laser energy dose about 4× higher than LED illumination (Figure 5C). Thus, by changing the energy dose for activating the optogenetic system, a controlled expression of BDNF is possible.

For future in vivo applications, the required spatial precision and light delivery method will have to be considered. Gallium nitride (GaN) LED has the advantage of a more compact footprint, such as in a µLED format integrated into flexible substrates [21, 22, 23]. Current research is ongoing in this field to improve the number of available addressable LEDs [24], producing stable thermo‐mechanical properties [25], and reducing losses due to the Lambertian LED emission profiles. On the other hand, lasers are excellent candidates as external light sources for applications where spatially localized stimulation is a priority. Lasers can be coupled to optical fibers for light delivery in the cochlea [21, 26] and focused to a small micron‐sized spot for single‐cell activation in the inner ear using miniaturized focusing optics. Overall, both LED and laser exhibit optical properties sufficient as an optogenetic switch. Despite the differences in spectral properties, with LEDs having a broader spectral bandwidth (447 ± 15 nm), than the laser used (473 ± 0.03 nm), both systems have shown robust light activation of CRY2/CIB interaction to regulate transcription and induce BDNF expression in mammalian cells without apparent phototoxicity effects of different light intensities.

### 2.6 Neurotrophic Effect of Light‐induced BDNF on Rat SGNs

Supernatant from light‐treated HEK293 cells was analyzed by Western blotting to confirm the presence of mature BDNF, as the precursor molecule proBDNF shows partial antagonistic effects on neuronal growth and survival. A prominent band at about 12 kDa was found, indicating mature BDNF. The precursor molecule proBDNF could not be detected with certainty (Figure S5).

**FIGURE 6:**
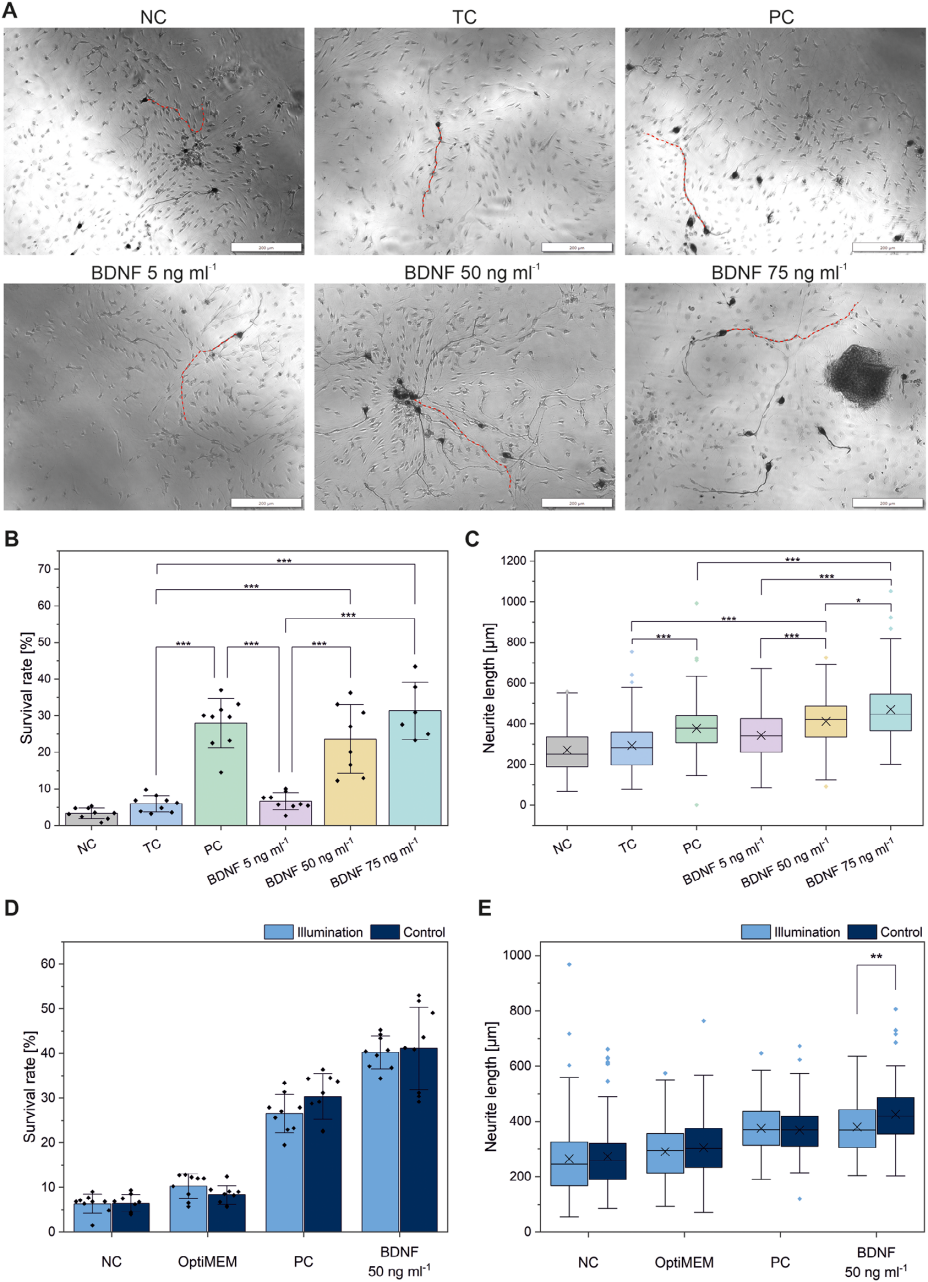
Neurotrophic effect of light‐induced BDNF on rat SGNs. (A) Representative images of rat SGNs (one neuron counted as survived exemplary traced in red), stained with DAB and treated with vehicle or supernatants of optogenetically activated HEK293 cells for two days, containing variable BDNF, quantified using ELISA. Scale bar 200 µm. (B) Diagram showing the mean survival rate of SGNs treated with HEK293 supernatants containing variable BDNF‐concentrations (mean ± sd, *N* = 3, *n* = 2–3, *p ≤ 0.05, **p ≤ 0.01, ***p ≤ 0.001, one‐way ANOVA with Tukey). (C) Median neurite length of SGNs treated with HEK293 supernatants containing variable BDNF‐concentrations (cross stands for mean value, *N* = 3, *n* = 2–3 (up to 135 counted neurites, Table S1), *p ≤ 0.05, **p ≤ 0.01, ***p ≤ 0.001, one‐way ANOVA with Tukey). llumination with blue light had no effect on (D) survival rate or (E) neurite length, except in the sample treated with optogenetically induced BDNF (50 ng mL^−1^), tested versus a non‐illuminated control of SGNs (cross stands for mean value, *N* = 3, *n* = 3). SGNs were illuminated for 12 h with LED light (20 s on/60 s off, 0.8 mW cm^−2^, 465 nm). NC = negative control with SGN culture medium only, OptiMEM = control with OptiMEM medium, PC = positive control with commercially available 50 ng mL^−1^ BDNF in PBS (+0.1% BSA), HEK293 supernatants in OptiMEM containing BDNF concentrations as indicated, TC = transfection control with supernatants of HEK293 cells optogenetically producing luciferase instead of BDNF.

To evaluate the biological activity of optogenetically produced BDNF, we added relevant concentrations to rat neonatal SGNs. Therefore, cell supernatants of light‐induced optogenetically manipulated HEK293 cells were added to freshly isolated neonatal rat SGNs in a 1:2 dilution with serum‐free SGN medium to obtain BDNF protein concentration levels of 5, 50, and 75 ng mL^−1^. After 2 days of cultivation SGNs were stained using DAB‐staining and underwent microscopic analyses (Figure 6A). The addition of HEK293 cell supernatant with 50 ng mL^−1^ BDNF led to a significantly elevated survival rate of SGNs compared to the negative control (NC) with SGN culture medium and the transfection control (TC) with supernatant from transfected HEK293 cells optogenetically producing luciferase instead of BDNF (Figure 6B). Additional controls are summarized in Table S1. The effect was comparable to the positive control (PC) of commercially available BDNF with the same concentration. The addition of HEK supernatants with an increased BDNF concentration of 75 ng mL^−1^ led to a further increase of the survival rate. Furthermore, the neurite length increased significantly by 99.3 µm and 158.1 µm under the influence of optogenetically induced BDNF in a concentration of 50 and 75 ng mL^−1^ compared to the TC. In contrast, the addition of the supernatant with a low BDNF level of 5 ng mL^−1^ had no significant effect on the survival rate and neurite length. The background expression of the optogenetic system remained below this threshold with a maximum concentration of 2.5 ± 1.1 ng mL^−1^ (see Figure 6C) and thus also had no effect on the SGNs. Here it has to be mentioned that according to Scheper et al., endogenously produced BDNF may have effects in smaller concentrations due to a comparably higher biological activity [27].

While other researchers have observed transcriptional changes and reduced viability of primary neuronal cells after blue light illumination, C. G. Duke et al. were able to show that these effects are caused by phototoxic interactions with neuronal culture media [28]. In general, it is assumed that drawing conclusions from ex vivo experiments with neuronal cultures to the in vivo situation can be problematic, as the neuronal cell culture generally does not reflect the physiological situation. Nevertheless, we investigated phototoxicity ex vivo in neonatal rat SGNs. Here we could show that the illuminance and wavelength of blue light (up to 0.8 mW cm^2^ and 465 nm) used in this work had no effect on the overall survival rate of SGNs over a period of 12 h (Figure 6D). However, the neurite length under blue light was significantly less pronounced compared to a non‐illuminated control in an approach with addition of optogenetically induced BDNF (from the HEK light induction approach). Interestingly, this light effect was not observed in any of the other controls (Figure 6E). In all samples treated with optogenetically induced BDNF, a growth‐promoting effect on SGN neurons was shown compared to non‐treated samples. Thus, overall, we could not detect a sustained phototoxic effect on SGNs, and we consider this even less likely under in vivo conditions due to the natural microenvironment of the neurons. Furthermore, under in vivo conditions, the light intensity and duration of the optogenetic approach would be lower, and light scattering can be reduced by using a focused laser beam. Even a repetitive illumination cycle for 12 h over a total of 3 d showed no changes in the survival rate in an exemplary experiment (Figure S6).

Overall, our results show that light induction of human cells for neuroprotection was successful and leads to different BDNF levels in a light dose‐dependent manner (Figures 2B/5C). The use of optogenetic applications in the inner ear requires an implantable light source. This is particularly true for blue light systems, which have a low penetration depth into the tissue, but this appears to be feasible by implantation of biocompatible laser‐coupled optical fibers [21, 29].

Moreover, optogenetic therapies, as opposed to consecutive gene expression with a strong promoter, allow for a tight and reversible control of BDNF release and timing, and thus control of BDND dosage is enabled. This is important because decreased BDNF levels are associated with mental illness in patients [30, 31], whereas, overexpression of BDNF leads to abnormal activation of neuronal circuits, resulting in learning and memory impairments [32]. Increased BDNF expression should, therefore, only occur within a certain time window and BDNF should only be overexpressed in a controlled manner until the desired effect has been achieved—that is, until the SGNs have recovered, as shown in this study—to avoid uncontrolled growth. A recent publication reports the potential therapeutic effect of increasing BDNF in various neurodegenerative diseases [33]. However, the authors explicitly postulate that the use of BDNF therapy must be strategically planned to ensure that it is effective and safe and administered in an appropriate volume and spatial context. The optogenetic approach presented here is thus dedicated to this goal.

A limitation of this study is the long illumination time of 6 h to achieve concentrations of 50 ng mL^−1^. In addition, maximum BDNF protein expression is not reached until 12 h after the end of the actual illumination. We cannot say, whether this is due to the slow reversibility of the CRY2/CIB system or to the stability of the mRNA. However, the maximum BDNF concentrations achieved here are not required for therapeutic applications in the inner ear and in vivo experiments. For example, Chikar et al. measured effective BDNF levels in the cochlear fluid of as low as 3.7 ± 2.95 ng mL^−1^ after inoculation of adenovirus constructs for consecutive BDNF overexpression in the cochlea of guinea pigs, showing the effect of an increase in the number of surviving spiral ganglion cells compared to an empty vector control [34]. Therefore, the illumination time and intensity will be shorter and lower, respectively, than the values used here. In summary, this study shows the characterization of the CRY2/CIB system, the illumination setup and the readout of the SGN experiment for optogenetic applications in the inner ear and serves as preparation for future in vivo tests.

## 3 Conclusion and Outlook

This study shows for the first time a new pharmacological principle for neurotrophic factor release using optogenetics. It demonstrates that optogenetics is applicable for BDNF gene and protein expression in a standardized and controllable manner using LEDs as well as lasers as potential light sources. Furthermore, it would be easy to replace BDNF with other neurotrophins as they are needed. Thus, it appears feasible, that optogenetic approaches once transferred to cells suitable as a low‐immunogenic cell therapeutic measure, such as mesenchymal stem/stromal cells (MSCs), can reach the level of clinical applicability. MSCs have high modulatory flexibility, plasticity, and low immunogenicity that renders them suitable candidates for therapeutic approaches [35] and cells such as hair cells, neurons, and spiral bands are promising targets for MSC‐mediated regenerative therapies [36]. In a clinical setting, these cells could be transfected ex vivo with viral vectors, allowing high transfection efficiency and better control of the plasmid ratio than direct vector application in the cochlear niche, and fixed on a suitable scaffold for implantation. For optical stimulation in the inner ear, microscale light delivery systems could be introduced via the surgical channel for the CI electrode to reduce surgical effort, with the optogenetically activatable cell scaffold and a laser‐based [37, 38] or LED [39, 40] light source implanted near the target site. We suggest that a laser beam is used to avoid the possible scattered radiation of an LED and thus undesirable side effects. Although pre‐clinical studies on optical stimulation were already performed in rodent models, for clinical translation, long‐term evaluation of material compatibility and stability as well as the thermal effects and changes in neural cell biology are still pending.

Furthermore, substantial research efforts are currently given to developing optical cochlea implants [22, 41]; hence, optogenetics for neurotrophic factor release can be highly compatible as a concomitant therapy.

## 4 Material and Methods

### 4.1 Molecular Biology

The plasmids encoding CRY‐GalΔDD (92035), CIB‐VP64 (92037), and pGL2‐GAL4‐UAS‐Luc (33020), kindly provided by Dr. C. Tucker, were purchased from addgene (Watertown, USA) and described in [42] and [43], respectively. For BDNF expression the plasmid pGL2‐Gal4‐UAS‐BDNF was generated. pGL2‐GAL4‐UAS‐Luc was digested with HindIII and ClaI (New England Biolabs, Frankfurt am Main, Germany) to remove luciferase. BDNF was amplified from a plasmid kindly provided by the working group of Andrea Hoffmann (Hannover Medical School, Hannover, Germany) using the primers 5′ GCATGAAAGCTTATGACCATCCTTTTCCTTACTATG and 5′ GACCATTAAAAGGGGAAGATAGATCGATGCATGA (Invitrogen by Thermo Fisher, Waltham, USA). Subsequently, the vector and insert were ligated with T4 DNA ligase (Thermo Fisher, Waltham, USA). Successful cloning was confirmed by sequencing (Microsynth Seqlab, Göttingen, Germany).

### 4.2 Cell Culture

HEK 293 cells (DSMZ (German Collection of Microorganisms and Cell Cultures, Braunschweig, Germany) were expanded in Dulbecco's Modified Eagles’ Medium (DMEM, D5864 high glucose, Sigma Aldrich, St. Louis, USA) supplemented with 10% fetal calf serum (FCS) (Biochrom, Berlin, Germany) and 1% penicillin/streptomycin (p/s) (Biochrom, Berlin, Germany) at 37°C and 5% CO_2_ and harvested by Accutase treatment (Merck KGaA, Darmstadt, Germany).

### 4.3 Ethics Statement for Isolation of SGNs from Neonatal Rats

The experiments were conducted in accordance with the German Animal Welfare Act and the European Directive 2010/63/EU for the protection of animals used for experimental purposes. The use of animals for tissue extraction (§4 German Animal Welfare Act) is registered (2018/215 and 2023/251) with the local authorities (Lower Saxony State Office for Consumer Protection and Food Safety [LAVES], Oldenburg, Germany) and the number of used animals reported annually. All rats were bred for research study purposes. A breeding stock was supplied by Charles River (Charles River, Sulzfeld, Germany) and housed with their litters in the facilities of the licensed Institution of Laboratory Animal Science of the Hannover Medical School.

### 4.4 Rat SGN Cell Culture

Neonatal Sprague–Dawley rats (postnatal days 3–5) were sacrificed by rapid decapitation before any experimentation. The following dissection of the cochleae as well as the mechanical and enzymatic dissociation of the spiral ganglia was performed according to a previously described protocol [44], resulting in mixed cell cultures of neurons, fibroblasts, and glial cells. Dissociated cells were seeded at a density of 1 × 10^4^ cells per well in a 96‐well plate coated with poly‐D/L‐ornithine (0.1 mg mL^−1^, Sigma‐Aldrich, Darmstadt, Germany) and laminin (0.01 mg mL^−1^, Gibco by Life Technologies, Carlsbad, USA). Cells were cultivated in equal volumes (50 µL) of completed SGN serum‐free medium (Panserin 401, PAN Biotech, Aidenbach, Germany), and the supernatants of the HEK293 cells (cultivated in serum‐free OptiMEM; harvested and stored at −20°C) after illumination experiments. Panserin 401 was supplemented with HEPES (23.4 mM, Invitrogen, Thermo Fisher Scientific, Darmstadt, Germany), glucose (0.15%, Braun AG, Melsungen, Germany), penicillin (30 U mL^−1^, Sigma‐Aldrich), N2 supplement (0.1 µg mL^−1^, Invitrogen) and insulin (8.7 µg mL^−1^, Sigma‐Aldrich). The following controls were included in all experiments: a SGN medium control (Panserin 401 complete medium), a HEK293 medium control (1:2 OptiMEM and Panserin 401 complete), a positive control (PBS with 0.1% BSA with 50 ng mL^−1^ exogenous recombinant BDNF additive), and a seeding control fixated after 4 h of cultivation served as reference. To evaluate the neurotrophic effect of light‐induced BDNF on SGNs, the cells were fixed after 48 h of cultivation at 37°C and 5% CO_2_, with a 1:2 methanol (Carl Roth, Karlsruhe, Germany) and acetone (J.T. Baker, Deventer, Netherlands) solution for 10 min and washed with PBS (Gibco by Life Technologies). To evaluate the neurotrophic effects of SGNs during direct blue‐light treatment, the cells were seeded and treated as mentioned above. But, after 24 h, the first treatment with blue‐light was performed for 12 h followed by a further incubation period of 12 h in dark after which the cells were fixed (total incubation time 48 h). For the repetitive illumination over a total of 3 d, also a pre‐incubation in with the light‐induced supernatants and respective controls for 24 h was performed, followed by an illumination cycle of 12 h illumination and 12 h incubation in the dark for three times (total incubation of 96 h/4 d). Media were changed after two days. For all illumination experiments, the respective identical control plate was kept in the dark during the illumination cycle and was also fixed accordingly.

### 4.5 Evaluation of the Survival Rate and Neurite Length of SGNs

SGNs were identified by staining with a monoclonal mouse 200 kDa‐neurofilament antibody (#NCL‐L‐NF200‐N52, Novocastra, Leica Biosystems, Wetzlar, Germany) as a neuron‐specific marker. Therefore, fixed cells were incubated with the primary neurofilament antibody for 1 h at 37°C, washed with PBS, and incubated with a secondary biotinylated anti‐mouse antibody (Vector Laboratories Inc., Burlingame, USA) for 30 min at RT. After another washing step with PBS, ABC complex solution (Vectastain Elite ABC‐Kit, Vector Laboratories Inc.) was added for 30 min according to the manufacturer's protocol. The staining was visualized by adding diaminobenzidine (Peroxidase Substrate Kit DAB, Vector Laboratories Inc.) for 10–14 min at RT, followed by a last washing step with PBS. Imaging of cells was performed with an inverted microscope (IX83, Olympus, Hamburg, Germany). SGNs were counted as survived when the neurite length amounted to at least three times the cell soma diameter [45]. The survival rate was calculated by relating the number of survived neurons per well to the seeding density after 4 h of cultivation. Furthermore, the length of up to the five longest neurites per image was measured with ImageJ based on five images per well (Table S1).

### 4.6 Gene Transfection and Illumination

The transfection of HEK293 cells was performed 24 h after seeding 12 × 10^3^ cells per well in a 96‐well plate. DreamFect Gold was used for transfection, according to the manufacturer's instruction (Oz biosciences, Marseille, France). Three plasmids CRY‐GalΔDD, CIB‐VP46, and pGL2‐Gal4‐UAS‐Luc or pGL2‐Gal4‐UAS‐BDNF were co‐transfected at a ratio of 1:1:X (X: 1–8). If not stated otherwise, the total amount of DNA was 0.75 µg per four wells. All samples were wrapped in aluminum foil, immediately after transfection, and incubated for another 24 h. For light‐treated samples, blue light was applied using either LED (20 s pulse delivered every 80 s over 12 h, 0.8 mW cm^−2^) as a light source or laser (0.6, 1.9, 3.5 mW over 6 h). Samples were analyzed 48 h after transfection to allow protein expression. Non‐treated samples were kept in the dark for the duration as a control.

### 4.7 LED Setup

A custom LED illumination chamber was developed for the cell illumination approach in cooperation with the working group of Holger Blume (Institute for Microelectronic Systems, Leibniz University Hannover, Germany; see Figure S2). It consists of six blue LEDs (OVA‐1063) with a wavelength of 465 nm, positioned under the ceiling of the opaque illumination chamber. All LEDs are driven through a TLC5940 (Texas Instruments), a specialized LED‐driver chip, which is controlled by a ATMEGA Microcontroller. The desired configuration, that is, light intensity (64 discrete intensity levels) and illumination duration can be regulated through customized software from a connected tablet. An integrated camera at the ceiling allowed verification of light treatment. A height‐adjustable stand was set to a defined working distance of 15 cm. Cell conditions during illumination were maintained using a mobile incubation chamber (Stage Top Incubation System [10720] containing a heating system [10918] and gas incubation system [11920], ibidi, Gräfeling, Germany). The LED power was measured using a power meter with a thermophilic sensor of 0.71 cm^2^ (919P‐003‐10, Newport, Darmstadt, Germany).

### 4.8 Laser Setup, Illumination Time, and Laser Control

The laser scanning setup utilizes a continuous wave laser emitting at a wavelength of 473 nm (Gem 473, Laser Quantum GmbH). A pair of plano‐convex lenses (*f* = 125.0 mm) collimates the beam which is directed to 2‐axis galvanometer mirrors. A 2‐inch plano‐convex lens (*f* = 100.0 mm) focuses the laser onto the well plate with a spot diameter of 100 µm. The galvanometer mirrors scan the laser spot on each well from top to bottom in a 2D meandering pattern. The laser power was measured using a power meter (PM100USB, Thorlabs, Newton, USA) at the well plate position. A motorized stage positions each well to the laser focus. To maintain the cells at 37°C, 5% CO_2_, the well plate was placed inside an incubation chamber (Stage Top Incubation System [10720] containing a heating system [10918] and a gas incubation system [11920], ibidi, Gräfeling, Germany) throughout the entire experiment.

Custom‐made software controlled the galvanometer mirrors and the motorized stage. The distance between wells and the well diameter was user‐defined in order to adapt the system to different types of well plates. Therefore, the required motion distance of the stage can be calculated automatically. After scanning an entire well, the motorized stage positions the well plate so that the next well coincides with the scanning area of the laser focus. For every experiment, four wells were illuminated for a duration of 6 h. For the laser scanning speed of 35 mm s^−1^, the total scanning time for the four wells including the return of the motorized stage to its initial position was measured to be 113 s, which sums up to about 191 line scans per well in 6 h.

### 4.9 Comparative Assessment of the Light Sources Used

To provide comparable illumination parameters for the LED and laser systems, we quantified the accumulated energy dose. For the LED setup, the calculation is performed using the relation:

(1) $$E_{\mathrm{dose}}=I\cdot \Delta t,$$

where *I* is the intensity and Δ*t* is the total illumination time. Light intensity under LED illumination is *I*  = 0.8 mW cm^−2^. For the LED system (Figure S2), a 20 s on/60 s off protocol (25% duty cycle), results in *E*_dose_ = 4.3 × 10^3^ mJ cm^−2^. To obtain the accumulated energy dose for the scanning laser setup, we first calculated the fluence (*F*) of the laser spot by using the relation [46]:

(2) $$F=\frac{P}{a\cdot \nu }\;$$

where *a* is the laser spot diameter, and ν the scanning speed. The laser power used for the experiments is 0.6, 1.9, and 3.3 mW that corresponds to calculated fluences of 17, 53, and 94 mJ cm^−2^, respectively. Multiplying the fluences with the number of line scans on each well leads to the accumulated energy dose of* *3.3 × 10^3^, 1.0 × 10^4^, 1.8 × 10^4^ mJ cm^−2^, which are ∼1×, 2×, and 4× of the LED energy dose for the entire 6 h duration of the experiment.

### 4.10 Cell Viability

The effect of blue light illumination on cell viability and proliferation was measured indirectly by the Cell Titer‐Blue (CTB) cell viability assay (Promega, Mannheim, Germany). Twenty‐four hours after the start of the illumination, the cell culture medium was removed and 100 µL CTB working solution (1:10 [v/v] dissolved in basal DMEM) was added to each well. After 4 h of incubation, CTB fluorescence was measured at an extinction wavelength of 544 nm and an emission wavelength of 590 nm using a microplate reader (Spectramax I3X, Molecular Devices, San José, USA). Values were normalized to the non‐illuminated control.

### 4.11 Inhibition of Secretion

BDNF secretion was inhibited with Brefeldin A treatment, an agent that induces retrograde transport from the Golgi apparatus to the endoplasmic reticulum. Six hours after illumination of HEK293 cells, OptiMEM was replaced with fresh medium containing 5 µg mL^−1^ Brefeldin A and the cells were incubated for another 6 h [47]. After a total of 12 h of illumination, supernatants were collected and BDNF concentration was measured by ELISA.

### 4.12 Luciferase Assay

Luciferase (Fluc) activity was measured 48 h after illumination with the Bright‐Glo Luciferase Assay System (Promega, Madison, USA) according to the manufacturer's protocol. The luminescence [relative light unit], measured with a microplate reader (Spectramax I3X, Molecular Devices, San José, USA), was quantified and converted to enzyme unit U using an external luciferase standard. Therefore, a serial dilution of the lyophilized luciferase with 11 U mg^−1^ (L9420, Sigma Aldrich, Taufkirchen, Germany) was prepared in 1 M Tris‐HCl with a pH of 7.5 (TRIS, 5429.1, Roth, Karlsruhe, Germany; HCl, 181021, AppliChem GmbH, Darmstadt, Germany) in the dark.

### 4.13 BDNF Assay

Quantification of BDNF in the supernatants was performed using the human BDNF ELISA Kit (RAB0026, Sigma Aldrich, Taufkirchen, Germany) according to the manufacturer's protocol. All supernatants were stored at −20°C for analysis. Supernatants of illuminated samples were diluted 1:25 to 1:500 and of control samples 1:5 in the Kit's 1X Assay Diluent B buffer.

### 4.14 Western Blot Analysis

HEK293 supernatants were stored at −20°C. A BDNF control was used from the BDNF ELISA Kit, diluted in the 1X Assay Diluent B buffer. Samples were mixed with Laemmli buffer and incubated for 10 min at 90°C. The proteins were separated on a 12% sodium dodecyl sulfate‐polyacrylamide gel electrophoresis and transferred to a polyvinylidene difluoride membranes for 90 min at 50 V (EasyPhor PAGE Mini Elektrophorese & HI Blotting, Biozym, Hessisch Oldendorf, Germany). The proteins were fixed with 5 % acetic acid for 5 min and afterwards, the membranes were blocked with 3% BSA in PBST for 90 min at RT and incubated with rabbit BDNF polyclonal antibody (1:1000, PA585730, Thermo Fisher, Germany) overnight at 4°C. Subsequently, the membranes were washed with PBST and incubated with peroxidase‐conjugated anti‐rabbit polyclonal antibody (1:10,000, STAR121P, Biorad) for 90 min at RT. Membranes were incubated with HRP substrate (Radiance Q, AC2101, Azure Biosystems, Dublin, USA) for 2 min and blot images were taken using chemiluminescence with the western blot imager (Azure c600, Azure Biosystems, Dublin, USA).

### 4.15 Real‐Time PCR Analysis

For quantitative real‐time polymerase chain reaction (qPCR), cells were lysed at different times (12, 24, 30, 48, 54, and 72 h) after transfection and the lysate was stored at −80°C for further preparation. RNA was isolated using the RNeasy Mini Kit (Qiagen, Hilden, Germany). Isolated RNA was normalized to a concentration of 25 ng µL^−1^ and was reverse‐transcribed to cDNA with RevertAid H Minus First Strand cDNA Synthesis Kit (Thermo Fisher, Waltham, USA) using random hexamers. qPCR was performed to measure gene transcript levels using the primers shown in Table S2. The housekeeping genes RPL4, PPIA, and B2 M served as reference genes. The thermal cycling conditions used on a real‐time PCR system (CFX384 Real‐time system C1000 touch, Biorad, Hercules, USA) with Sso Advanced Universal SYBR Green Supermix (Biorad, Hercules, USA) were 95°C for 2 min, following 40 cycles at 95°C for 20 s for denaturation, 56.1°C–63.4°C (depending on used primers) for 15 s for annealing, and 70°C for 10 s extension with real‐time data recording. The cycles were followed by a 2 min final extension at 70°C and melt curve recording (75°C–95°C). The specificity of amplification was monitored by melt curve analysis and through negative controls containing no cDNA. A pipetting robot (Epmotion 5073, Eppendorf, Hamburg, Germany) to minimize measurement inaccuracies pipetted all samples, references, master mixes, and standards. The relative quantification of the mRNA expression was determined using the Vandesompele method as described in [48], in quadruple, and the average cycle threshold (CT) was calculated. Non‐illuminated sample was set as the calibrator sample.

### 4.16 Statistical Analysis

Data were obtained from *N* independent experiments with n replicates per condition as indicated in the respective legends and results were depicted as means ± standard deviation (sd). Levels of significance were analyzed using one‐way analysis of variance (ANOVA) with Tukey's post hoc test. Differences were considered significant at *p* ≤ 0.05. Significance levels were indicated with *p ≤ 0.05, **p ≤ 0.01, and ***p ≤ 0.001. Results for the survival rate of SGNs stimulated with BDNF were gained out of 6–9 SGN samples per group. For the determination of the neurite length, up to 129 neurons were tracked per group (Table S2). Here, a non‐parametric statistical test (Mann–Whitney‐Test) was performed with a level of significance of minimally *p* ≤ 0.05. Significance levels were indicated with *p ≤ 0.05, **p ≤ 0.01, and ***p ≤ 0.001.

## Author Contributions

Conceptualization: Sina Christoffers and Cornelia Blume. Formal analysis: Sina Christoffers. Funding acquisition: Cornelia Blume. Investigation: Sina Christoffers, Nina Wichert, Elena Wiebe, Madeleine Goblet, Jennifer Harre, and Odett Kaiser. Methodology: Sina Christoffers, Nina Wichert, Maria Leilani Torres‐Mapa, Jennifer Harre, and Odett Kaiser. Software: Marc‐Nils Wahalla, and Maria Leilani Torres‐Mapa. Project administration: Cornelia Blume, Holger Blume, Alexander Heisterkamp, Athanasia Warnecke. Writing–original draft preparation: Sina Christoffers. Writing–review and editing: Cornelia Blume, Maria Leilani Torres‐Mapa, Jennifer Harre, and Odett Kaiser.

## Conflicts of Interest

The authors declare no conflicts of interest.

## Supporting information



Supporting Information

## Data Availability

Technical data on the laser (M. L. T‐M), LED setup (M‐N. W.), or experimental data (S. C.) are available on request from the respective authors.

## References

[1] M. Häusser , “Optogenetics: The Age of Light,” Nature Methods 11, no. 10 (2014): 1012–1014, 10.1038/nmeth.3111. 25264778

[2] N. Wichert , M. Witt , C. Blume , and T. Scheper , “Clinical Applicability of Optogenetic Gene Regulation,” Biotechnology and Bioengineering 118, no. 11 (2021): 4168–4185, 10.1002/bit.27895. 34287844

[3] M. J. Kennedy , R. M. Hughes , L. A. Peteya , J. W. Schwartz , M. D. Ehlers , and C. L. Tucker , “Rapid Blue‐Light–Mediated Induction of Protein Interactions in Living Cells,” Nature Methods 7, no. 12 (2010): 973–975, 10.1038/nmeth.1524. 21037589 PMC3059133

[4] J. R. Quejada , S.‐H. E. Park , D. W. Awari , et al., “Optimized Light‐Inducible Transcription in Mammalian Cells Using Flavin Kelch‐Repeat F‐box1/GIGANTEA and CRY2/CIB1,” Nucleic Acids Research 45, no. 20 (2017): e172, 10.1093/nar/gkx804. 29040770 PMC5714181

[5] M. Yazawa , A. M. Sadaghiani , B. Hsueh , and R. E. Dolmetsch , “Induction of Protein‐Protein Interactions in Live Cells Using Light,” Nature Biotechnology 27, no. 10 (2009): 941–945, 10.1038/nbt.1569. 19801976

[6] L. Duan , J. Hope , Q. Ong , et al., “Understanding CRY2 Interactions for Optical Control of Intracellular Signaling,” Nature Communications 8, no. 1 (2017): 547, 10.1038/s41467-017-00648-8. PMC5601944 28916751

[7] L. R. Polstein and C. A. Gersbach , “Light‐Inducible Spatiotemporal Control of Gene Activation by Customizable Zinc Finger Transcription Factors,” Journal of the American Chemical Society 134, no. 40 (2012): 16480–16483, 10.1021/ja3065667. 22963237 PMC3468123

[8] R. Khanna , E. Huq , E. A. Kikis , B. Al‐Sady , C. Lanzatella , and P. H. Quail , “A Novel Molecular Recognition Motif Necessary for Targeting Photoactivated Phytochrome Signaling to Specific Basic Helix‐Loop‐Helix Transcription Factors,” Plant Cell 16, no. 11 (2004): 3033–3044, 10.1105/tpc.104.025643. 15486100 PMC527196

[9] K. Müller , R. Engesser , S. Metzger , et al., “A Red/Far‐Red Light‐Responsive Bi‐Stable Toggle Switch to Control Gene Expression in Mammalian Cells,” Nucleic Acids Research 41, no. 7 (2013): e77, 10.1093/nar/gkt002. 23355611 PMC3627562

[10] N. Noda and T. Ozawa , “Light‐Controllable Transcription System by Nucleocytoplasmic Shuttling of a Truncated Phytochrome B,” Photochemistry and Photobiology 94, no. 5 (2018): 1071–1076, 10.1111/php.12955. 29893404

[11] M. Russ , A. K. Ehret , M. Hörner , et al., “Opto‐APC: Engineering of Cells That Display Phytochrome B on Their Surface for Optogenetic Studies of Cell‐Cell Interactions,” Frontiers in Molecular Biosciences 10 (2023): 1143274, 10.3389/fmolb.2023.1143274. 36936981 PMC10016228

[12] Y. Zhou , D. Kong , X. Wang , et al., “A Small and Highly Sensitive Red/Far‐Red Optogenetic Switch for Applications in Mammals,” Nature Biotechnology 40, no. 2 (2022): 262–272, 10.1038/s41587-021-01036-w. 34608325

[13] J. Jang , K. Tang , J. Youn , et al., “Engineering of Bidirectional, Cyanobacteriochrome‐Based Light‐Inducible Dimers (BICYCL)s,” Nature Methods 20, no. 3 (2023): 432–441, 10.1038/s41592-023-01764-8. 36823330

[14] K. Müller , M. D. Zurbriggen , and W. Weber , “Control of Gene Expression Using a Red‐ and Far‐Red Light–Responsive Bi‐Stable Toggle Switch,” Nature Protocols 9, no. 3 (2014): 622–632, 10.1038/nprot.2014.038. 24556785

[15] Y. Uda , H. Miura , Y. Goto , et al., “Improvement of Phycocyanobilin Synthesis for Genetically Encoded Phytochrome‐Based Optogenetics,” ACS Chemical Biology 15, no. 11 (2020): 2896–2906, 10.1021/acschembio.0c00477. 33164485

[16] A. Acheson , J. C. Conover , J. P. Fandl , et al., “A BDNF Autocrine Loop in Adult Sensory Neurons Prevents Cell Death,” Nature 374, no. 6521 (1995): 450–453, 10.1038/374450a0. 7700353

[17] S. Bathina and U. N. Das , “Brain‐Derived Neurotrophic Factor and Its Clinical Implications,” Archives of Medical Science: AMS 11, no. 6 (2015): 1164–1178, 10.5114/aoms.2015.56342. 26788077 PMC4697050

[18] E. J. Huang and L. F. Reichardt , “Neurotrophins: Roles in Neuronal Development and Function,” Annual Review of Neuroscience 24 (2001): 677–736, 10.1146/annurev.neuro.24.1.677. PMC2758233 11520916

[19] P. Deng , J. D. Anderson , A. S. Yu , G. Annett , K. D. Fink , and J. A. Nolta , “Engineered BDNF Producing Cells as a Potential Treatment for Neurologic Disease,” Expert Opinion on Biological Therapy 16, no. 8 (2016): 1025–1033, 10.1080/14712598.2016.1183641. 27159050 PMC5762114

[20] R. S. Duman , S. Deyama , and M. V. Fogaça , “Role of BDNF in the Pathophysiology and Treatment of Depression: Activity‐Dependent Effects Distinguish Rapid‐Acting Antidepressants,” European Journal of Neuroscience 53, no. 1 (2021): 126–139, 10.1111/ejn.14630. 31811669 PMC7274898

[21] A. Dieter , C. J. Duque‐Afonso , V. Rankovic , M. Jeschke , and T. Moser , “Near Physiological Spectral Selectivity of Cochlear Optogenetics,” Nature Communications 10, no. 1 (2019): 1962, 10.1038/s41467-019-09980-7. PMC6488702 31036812

[22] A. Dieter , E. Klein , D. Keppeler , et al., “µLED‐Based Optical Cochlear Implants for Spectrally Selective Activation of the Auditory Nerve,” EMBO Molecular Medicine 12, no. 8 (2020): e12387, 10.15252/emmm.202012387. 32596983 PMC7411546

[23] C. Goßler , C. Bierbrauer , R. Moser , et al., “GaN‐Based Micro‐LED Arrays on Flexible Substrates for Optical Cochlear Implants,” Journal of Physics D: Applied Physics 47, no. 20 (2014): 205401, 10.1088/0022-3727/47/20/205401.

[24] E. Klein , C. Gossler , O. Paul , U. T. Schwarz , and P. Ruther , “High‐Yield Indium‐Based Wafer Bonding for Large‐Area Multi‐Pixel Optoelectronic Probes for Neuroscientific Research,” Journal of Micromechanics and Microengineering 29, no. 9 (2019): 95006, 10.1088/1361-6439/ab2a53.

[25] E. Klein , C. Gossler , O. Paul , and P. Ruther , “High‐Density µLED‐Based Optical Cochlear Implant With Improved Thermomechanical Behavior,” Frontiers in Neuroscience 12 (2018): 659, 10.3389/fnins.2018.00659. 30327585 PMC6174235

[26] N. Kallweit , P. Baumhoff , A. Krueger , et al., “Optoacoustic Effect Is Responsible for Laser‐Induced Cochlear Responses,” Scientific Reports 6 (2016): 28141, 10.1038/srep28141. 27301846 PMC4908384

[27] V. Scheper , J. Schwieger , A. Hamm , T. Lenarz , and A. Hoffmann , “BDNF‐Overexpressing Human Mesenchymal Stem Cells Mediate Increased Neuronal Protection in Vitro,” Journal of Neuroscience Research 97, no. 11 (2019): 1414–1429, 10.1002/jnr.24488. 31257632 PMC6772136

[28] C. G. Duke , K. E. Savell , J. J. Tuscher , R. A. Phillips , and J. J. Day , “Blue Light‐Induced Gene Expression Alterations in Cultured Neurons Are the Result of Phototoxic Interactions With Neuronal Culture Media,” eNeuro 7, no. 1 (2020): 1–8, 10.1523/ENEURO.0386-19.2019. PMC6946540 31879366

[29] A. Agarwal , X. Tan , Y. Xu , and C.‐P. Richter , “Channel Interaction During Infrared Light Stimulation in the Cochlea,” Lasers in Surgery and Medicine 53, no. 7 (2021): 986–997, 10.1002/lsm.23360. 33476051 PMC8787864

[30] H. Soloey‐Nilsen , K. Nygaard‐Odeh , M. G. Kristiansen , et al., “Association Between Brain‐Derived Neurotropic Factor (BDNF), High‐Sensitivity C‐Reactive Protein (hs‐CRP) and Psychiatric Symptoms in Medicated and Unmedicated Patients,” BMC Psychiatry [Electronic Resource] 22, no. 1 (2022): 84, 10.1186/s12888-022-03744-2. 35114967 PMC8815216

[31] C. S. Weickert , C. H. Lee , R. K. Lenroot , et al., “Increased Plasma Brain‐Derived Neurotrophic Factor (BDNF) Levels in Females with Schizophrenia,” Schizophrenia Research 209 (2019): 212–217, 10.1016/j.schres.2019.04.015. 31088701

[32] S. D. Croll , C. Suri , D. L. Compton , et al., “Brain‐Derived Neurotrophic Factor Transgenic Mice Exhibit Passive Avoidance Deficits, Increased Seizure Severity and in Vitro Hyperexcitability in the Hippocampus and Entorhinal Cortex,” Neuroscience 93, no. 4 (1999): 1491–1506, 10.1016/S0306-4522(99)00296-1. 10501474 PMC2504500

[33] K. F. Azman and R. Zakaria , “Recent Advances on the Role of Brain‐Derived Neurotrophic Factor (BDNF) in Neurodegenerative Diseases,” International Journal of Molecular Sciences 23, no. 12 (2022): 6827, 10.3390/ijms23126827. 35743271 PMC9224343

[34] J. A. Chikar , D. J. Colesa , D. L. Swiderski , A. Di Polo , Y. Raphael , and B. E. Pfingst , “Over‐Expression of BDNF by Adenovirus With Concurrent Electrical Stimulation Improves Cochlear Implant Thresholds and Survival of Auditory Neurons,” Hearing Research 245, no. 1‐2 (2008): 24–34, 10.1016/j.heares.2008.08.005. 18768155 PMC2654221

[35] V. K. Mishra , H.‐H. Shih , F. Parveen , et al., “Identifying the Therapeutic Significance of Mesenchymal Stem Cells,” Cells 9, no. 5 (2020): 1145, 10.3390/cells9051145. 32384763 PMC7291143

[36] S. Kanzaki , M. Toyoda , A. Umezawa , and K. Ogawa , “Application of Mesenchymal Stem Cell Therapy and Inner Ear Regeneration for Hearing Loss: A Review,” International Journal of Molecular Sciences 21, no. 16 (2020): 5764, 10.3390/ijms21165764. 32796705 PMC7460950

[37] T. Mager , D. La Lopez de Morena , V. Senn , et al., “High Frequency Neural Spiking and Auditory Signaling by Ultrafast Red‐Shifted Optogenetics,” Nature Communications 9, no. 1 (2018): 1750, 10.1038/s41467-018-04146-3. PMC5931537 29717130

[38] A. C. Thompson , J. B. Fallon , A. K. Wise , S. A. Wade , R. K. Shepherd , and P. R. Stoddart , “Infrared Neural Stimulation Fails to Evoke Neural Activity in the Deaf Guinea Pig Cochlea,” Hearing Research 324 (2015): 46–53, 10.1016/j.heares.2015.03.005. 25796297

[39] S. Anpilov , Y. Shemesh , N. Eren , et al., “Wireless Optogenetic Stimulation of Oxytocin Neurons in a Semi‐Natural Setup Dynamically Elevates Both Pro‐Social and Agonistic Behaviors,” Neuron 107, no. 4 (2020): 644–655.e7, 10.1016/j.neuron.2020.05.028. 32544386 PMC7447984

[40] Z. Liu , C.‐H. Lin , B.‐R. Hyun , et al., “Micro‐Light‐Emitting Diodes with Quantum Dots in Display Technology,” Light, Science & Applications 9, no. 83 (2020), 10.1038/s41377-020-0268-1. PMC7214519 32411368

[41] C. Wrobel , A. Dieter , A. Huet , et al., “Optogenetic Stimulation of Cochlear Neurons Activates the Auditory Pathway and Restores Auditory‐Driven Behavior in Deaf Adult Gerbils,” Science Translational Medicine 10, no. 449 (2018): eaao0540, 10.1126/scitranslmed.aao0540. 29997248

[42] G. P. Pathak , J. I. Spiltoir , C. Höglund , et al., “Bidirectional Approaches for Optogenetic Regulation of Gene Expression in Mammalian Cells Using Arabidopsis Cryptochrome 2,” Nucleic Acids Research 45, no. 20 (2017): e167, 10.1093/nar/gkx260. 28431041 PMC5714224

[43] H. Nishio and M. J. Walsh , “Ccaat Displacement Protein/Cut Homolog Recruits G9a Histone Lysine Methyltransferase to Repress Transcription,” Proceedings of the National Academy of Sciences of the United States of America 101, no. 31 (2004): 11257–11262, 10.1073/pnas.0401343101. 15269344 PMC509191

[44] A. Warnecke , J. Harre , H. Staecker , et al., “Extracellular Vesicles From Human Multipotent Stromal Cells Protect Against Hearing Loss After Noise Trauma In Vivo,” Clinical and Translational Medicine 10, no. 8 (2020): e262, 10.1002/ctm2.262. 33377658 PMC7752163

[45] L. N. Gillespie , G. M. Clark , P. F. Bartlett , and P. L. Marzella , “BDNF‐Induced Survival of Auditory Neurons In Vivo: Cessation of Treatment Leads to Accelerated Loss of Survival Effects,” Journal of Neuroscience Research 71, no. 6 (2003): 785–790, 10.1002/jnr.10542. 12605404

[46] C. Wei , R. Yoshizaki , Y. Ito , et al., “High‐Speed Microgroove Processing of Glass by Expanding the High‐Temperature Region Formed by Transient and Selective Laser Absorption,” Optics Express 30, no. 18 (2022): 32280–32291, 10.1364/OE.464409. 36242293

[47] K. Oh‐Hashi , K. Tanaka , H. Koga , Y. Hirata , and K. Kiuchi , “Intracellular Trafficking and Secretion of Mouse Mesencephalic Astrocyte‐Derived Neurotrophic Factor,” Molecular and Cellular Biochemistry 363, no. 1‐2 (2012): 35–41, 10.1007/s11010-011-1155-0. 22120531

[48] J. Vandesompele , K. De Preter , F. Pattyn , et al., “Accurate Normalization of Real‐Time Quantitative RT‐PCR Data by Geometric Averaging of Multiple Internal Control Genes,” Genome Biology 3, no. 7 (2002): RESEARCH0034, 10.1186/gb-2002-3-7-research0034. 12184808 PMC126239

