# Supplementary material for: Blue Light‐Induced, Dosed Protein Expression of Active BDNF in Human Cells Using the Optogenetic CRY2/CIB System
Source: Biotechnol J. 2024 Dec 26;19(12):e202400384. doi: 10.1002/biot.202400384 (PMC11671672; doi:10.1002/biot.202400384)
Supplement: Supplementary file 1 — Supporting Information [file BIOT-19-e202400384-s001.pdf]

Supporting information

Blue light-induced protein expression of active BDNF in human cells using the optogenetic CRY2/CIB system

Sina Christoffers[1,2ǂ]*, Nina Wichert[ǂ1,2], Elena Wiebe[1,2], Maria Leilani Torres-Mapa[2,3], Madeleine Goblet[4], Jennifer Harre[4], Odett Kaiser[4], Marc-Nils Wahalla[2,5], Holger Blume[2,5], Alexander Heisterkamp[2,3], Athanasia Warnecke[2,4], Cornelia Blume[1,2]

[ǂ]Both authors contributed equally to this work

[1]*Institute of Technical Chemistry, Leibniz University Hannover, Callinstr. 3-5, 30167 Hannover, Germany*

[2]*Cluster of Excellence Hearing4all, Hannover, Germany*

[3] *Institute of Quantum Optics, Leibniz University Hannover, Welfengarten 1, 30167 Hannover, Germany*

[4] *Department of Otorhinolaryngology, Hannover Medical School, Carl-Neuberg-Str. 1, 30625 Hannover, Germany*

[5] *Institute of Microelectronic Systems, Leibniz University Hannover, Appelstr. 4, 30167 Hannover, Germany*

Keywords: gene expression, neurotrophins, BDNF, HEK293, spiral ganglion neurons, cochlea implant

Supplementary Tables S1-S2

Table S1: Detailed numbers of counted neurites of SGNs per treated group. Three wells were evaluated per group. Five images were taken per well and the five longest neurite lengths contained in an image were measured; the minimum criterion was a minimum neurite length of 3 times the soma average. All experiments were made in triplicates.

Group	Counted neurites
NK	47
PBS/BSA	38
PK	132
OptiMem	53
5 ng/ml	80
50 ng/ml	124
70 ng/ml	101

Table S2: qPCR primer, their annealing temperatures, sequences and resulting fragment lengths

NAME	PLASMID	ANNEALING TEMPERATURE	SEQUENCE	FRAGMEN T LENGTH
**B2M** FORWARD REVERSED	reference gene	56,1 °C	GCAAGGACTGGTCTTTCTAT GATGCTTGATTACATGTCTCG	140 bp
**BDNF** FORWARD REVERSED	BDNF	61,6 °C	CTCTTTCTGCTGGAGGAATACAA GCCGTTACCCACTCACTAATAC	131 bp
**CIB** FORWARD REVERSED	CIB	63,4 °C	CAAGTACCTCAATCCCACCTTT GTCGAAAGATAGCTGTCCATCTC	96 bp
**CRY** FORWARD REVERSED	CRY2	61,6 °C	GAATCCGTTATGCTTCCTCCTC CTCCAGCCCTAGTTCTTCAATC	96 bp
**LUC** FORWARD REVERSED	luciferase	61,6 °C	GTGTTGGGCGCGTTATTTATC TAGGCTGCGAAATGTTCATACT	97 bp
**PPIA** FORWARD REVERSED	reference gene	61,6 °C	CTCTGAGCACTGGAGAGAAAG CAGTGCCATTATGGCGTG	108 bp
**RPL4** FORWARD REVERSED	reference gene	60,6 °C	CAAAGGCAAAATGAGAAACCG GAAGGCCTTGATGATACCATTATC	88 bp

Supplementary Figures S1-S3

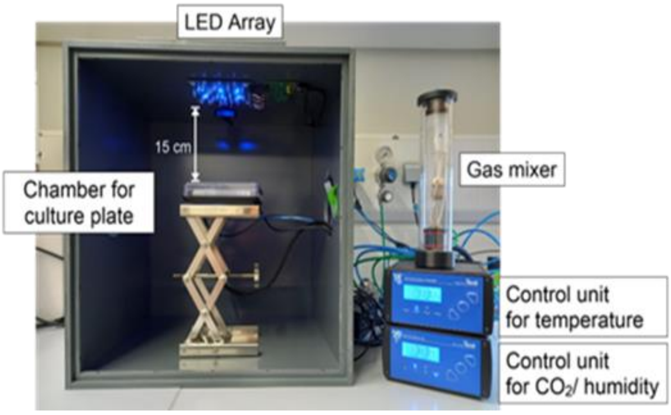

Figure S1: LED illumination chamber using a LED array consisting of six LEDs (465 nm) with adjustable intensity. Cells were cultivated in a mobile cultivation chamber with precise control of temperature, CO_2, and humidity.

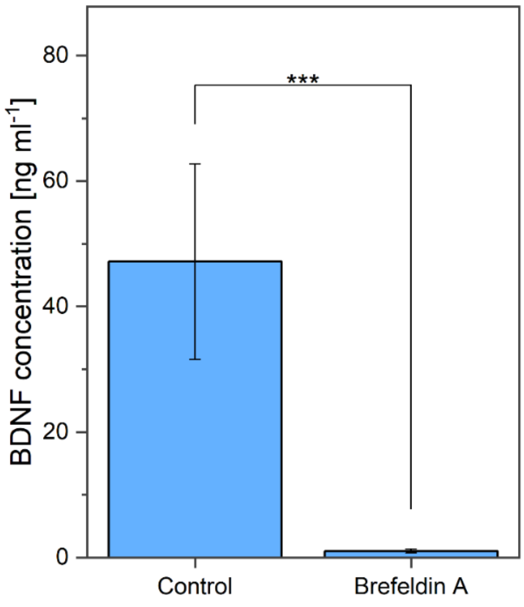

Figure S2: Inhibition of BDNF secretion. Six h after illumination of HEK293 cells, culture medium was replaced with fresh medium containing 5 µg ml^{-1} Brefeldin A and the cells were incubated for another 6 h. Supernatants were collected and BDNF concentration was measured by ELISA. Illuminated cells without Brefeldin A treatment served as control (mean, ± sd, N=3, n=4, ***p ≤ 0.001, one-way ANOVA with Tukey).

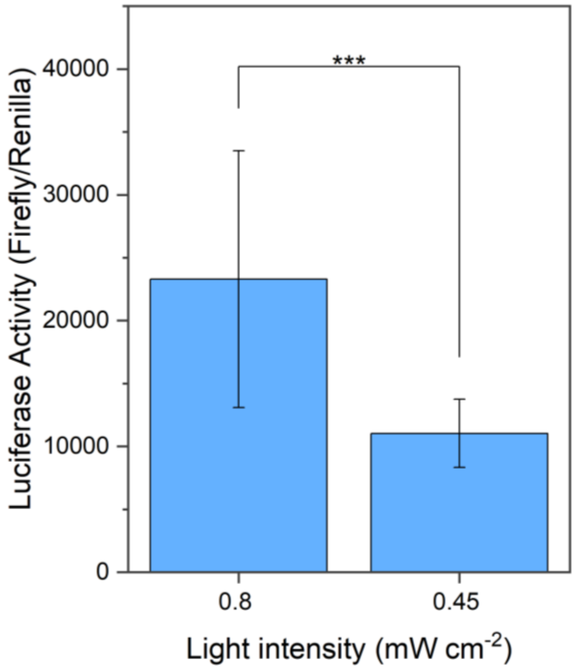

Figure S3: Influence of light intensity on luciferase activity after pulsed LED-illumination for 6 h (20 s on/60 s off, 465 nm), measured 48 h after transfection of HEK293 cells with a fluorimetric luciferase assay (Dual-Glo luciferase assay, promega). Reducing the light intensity by 50 % also reduces the luciferase activity by 50 % (mean, ± sd, N=3, n=4, ***p ≤ 0.001, one-way ANOVA with Tukey).

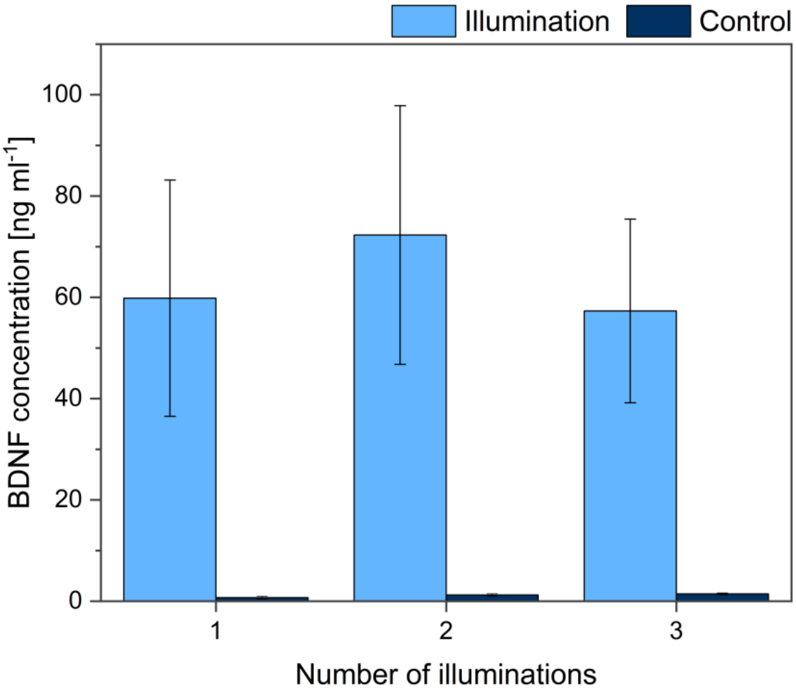

Figure S4: Repetitive activation of the optogenetic system. Pulsed LED illumination was applied on the same HEK293 cells on consecutive three days. After every illumination, the supernatant was exchanged with fresh cell culture medium and analyzed by ELISA (mean, ± sd, N=2, n=4).

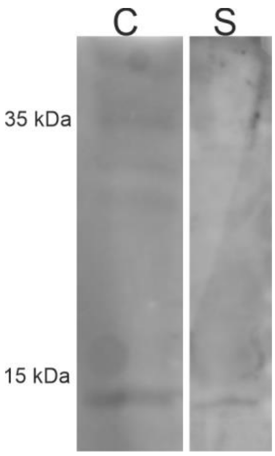

Figure S5: Western Blot analysis of illuminated HEK293 cell supernatant (S). Exogenous recombinant BDNF was used as a control (C). ProBDNF could not be sufficiently verified. Control shows some non-specific bands.

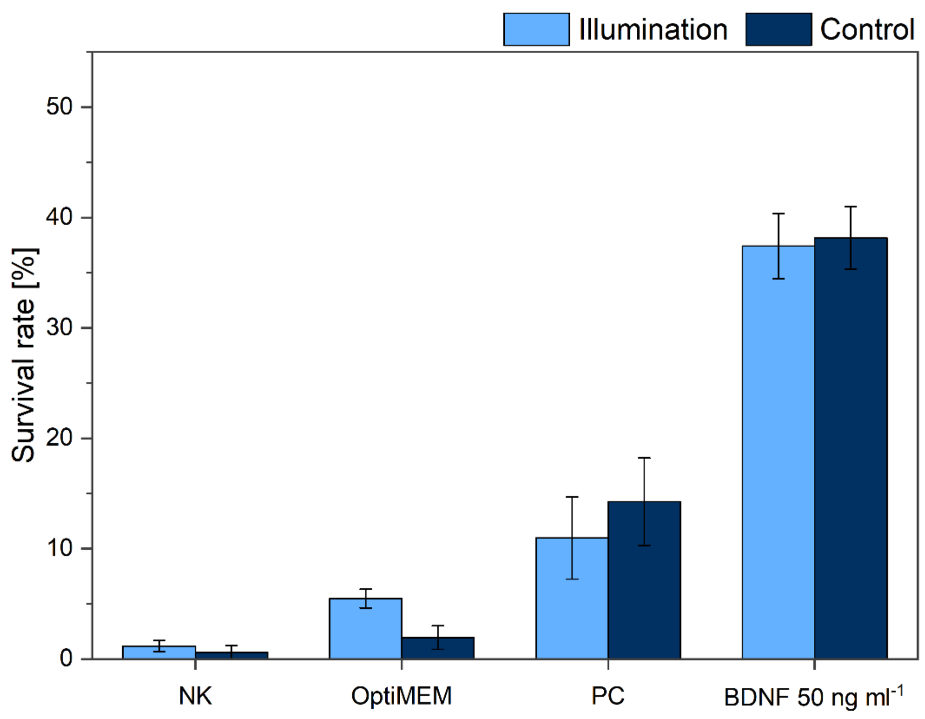

Figure S6: Repetitive illumination has no effect on the survival rate of SGNs. SGNs were illuminated over 12 h for 3d. Survival rate was calculated by normalizing the number of survived SGNs to a seeding control.
